# Validity and reliability of a smartphone-based photographic method for detection of dental caries in adults for use in teledentistry

**DOI:** 10.3389/froh.2025.1470706

**Published:** 2025-05-15

**Authors:** Victor F. Lamas-Lara, Manuel A. Mattos-Vela, Teresa A. Evaristo-Chiyong, Maria Eugenia Guerrero, Juan F. Jiménez-Yano, Dora N. Gómez-Meza

**Affiliations:** ^1^ Department of Dentistry, Policlinico Chincha, Essalud, Lima, Peru; ^2^ SAETA Research Group, Universidad Nacional Mayor de San Marcos, Lima, Peru; ^3^ Department of Medico Surgical Stomatology, Universidad Nacional Mayor de San Marcos, Lima, Peru

**Keywords:** dental caries, dental photography, teledentistry, data accuracy, validity and reliability

## Abstract

**Introduction:**

With the arrival of the COVID-19 pandemic, the role of teledentistry increased its need for implementation. In this sense, the study aimed to validate a smartphone-based remote photographic method for diagnosing dental caries in adults attending a polyclinic in Lima, Peru.

**Methods:**

A cross-sectional, descriptive research was conducted; 87 patients were selected, and 2020 teeth were evaluated. Each participant underwent a clinical diagnosis of dental caries by two trained and calibrated dentists, considering the diagnostic criteria of the WHO; during the same visit, after the clinical examination, a family member of the patient was instructed to take a photographic record with a cell phone, through a video. Five photographs of the dental arches were recorded, where the centering of images, resolution, and visualization of all the teeth were evaluated. The photographic evaluation was performed by two independent evaluators blinded to the visual evaluation performed, following the same criteria as the clinical visual evaluation. For the data analysis, Cohen's kappa index was determined for interexaminer reliability; sensitivity, specificity, and positive and negative predictive values were obtained.

**Results:**

Overall, high sensitivity: 90.19% [Interquartile 95% (CI): 88.23–92.16]; and specificity: 95.15% (95% CI: 93.83–96.47). The interexaminer agreement was almost perfect, with a kappa of 0.935 and 0.974 for clinical and photographic evaluation, respectively.

**Conclusions:**

It is concluded that the photographic method using a smartphone has demonstrated a satisfactory level of caries detection in adults.

## Introduction

Humanity has undergone great changes since the coronavirus disease (COVID-19) was declared a pandemic by the World Health Organization (WHO) on March 11, 2020 (1), and a series of measures were taken to reduce the spread of this outbreak, generating a strong demand at the level of the health sector. This situation led to adjustments and modifications in the provision of health care due to the restrictions inherent to the scarcity of human and material resources, as well as the need for social distancing (1, 2). In the dental area, most non-urgent procedures were suspended to reduce the risk of transmission in practice and prevent the spread of the virus, and only emergency treatment was maintained (3, 4). As time went by, the uncertainty regarding the course of the pandemic and the high demand for care forced health systems to find alternative solutions that could satisfy medical care and keep stakeholders away from exposure to SARS-CoV-2 (5).

Among these alternatives, teledentistry, an area of telemedicine that allows remote communication between dentists and patients by combining digital technology and clinical dentistry, has become very useful (2, 5–8). This area has presented in recent years great advances in informatics tools along with the availability of improved infrastructure for high bandwidth Internet access, creating unprecedented new routes for the use of remote health care applications (6, 9). Teledentistry as such has not been as widely used as in medicine; however, it can be incorporated into routine dental practice through a wide range of uses, such as teletriage (4), diagnosis (9, 10), treatment planning (11), consultation and follow-up in different specialties of dentistry (10), experiences that have been developing since the 1990s (5). It is essential to keep in mind that teledentistry encompasses various facets, and one of them focuses on telediagnosis. This process is materialized through the incorporation of intelligent systems and applications that work both in real-time and in data storage and sending format. This facilitates the efficient exchange of images and data, thus making it possible to identify various oral lesions. Among these, dental caries stands out as one of the most frequent (5, 6). In addition, teledentistry makes it possible to work with populations with limited access, facilitating the referral of patients to a dental consultant and their subsequent treatment and reducing waiting times and unnecessary trips (2, 6, 12).

On the other hand, among oral disorders, dental caries is an important public health problem (9, 13). For its evaluation, visual inspection is the most common technique; however, with advances in technology, smartphone cameras make it possible to obtain and transmit dental images (9). As specially designed oral digital cameras, smartphone cameras have zoom and flash functions and several manual settings that allow for easier capture of intraoral or extraoral images. In addition, it is important to consider the portability and accessibility features of these devices that may provide an effective means of capturing images in less time and be less intimidating for children (13–15). Thus, they can provide a simple, inexpensive, remotely applicable method of diagnosis of dental caries for both clinical and community use, which is very relevant in regions with varied geography. A photographic method such as the one described will make it possible to diagnose dental caries in people living in remote communities of Peru where access to health services is restricted. For this purpose, it will be necessary to send a few photographs and a diagnostic evaluation by a qualified professional. It is important to consider that this research provides the necessary scientific basis for using this method. Likewise, it is very useful in the context of community diseases, protecting the patient and health personnel.

This study made it possible to obtain an alternative diagnostic method in cases where the adult patient is unable to travel to a health facility, allowing savings for the patient in terms of costs and travel time, as well as for the hospital, facilitating care processes and saving personnel and resources.

The objective of this study was to validate the remote photographic method with the active participation of a family member of the patient, using a smartphone for the diagnosis of dental caries in adult patients attending a healthcare facility. This was achieved through the determination of the diagnostic accuracy and reliability of the photographic method.

## Methods

### Study design

A diagnostic accuracy study was conducted. The report was made following the “Standards for Reporting of Diagnostic Accuracy Studies” (STARD) (16, 17).

The present study, conducted with human beings, was governed by the principles and guidelines of the Declaration of Helsinki and was approved by the Ethics Committee of the Edgardo Rebagliati Martins National Hospital (1656_GRPR-ESSALUD-2022). The informed consent of the individuals was requested, and their participation in the clinical and photographic evaluation was voluntary and anonymous. There were no risks for the participants; the privacy and confidentiality of the personal information of the research subjects were protected, and the anonymity of participants was maintained for terms of publication. Furthermore, patients with detected cases were referred for appropriate care based on their needs, taking into account preventive aspects such as health promotion, dental brushing techniques, and healthy eating, as well as restorative procedures. All of this was in accordance with the clinical care protocol of ESSALUD (18).

### Population and sample

**Figure 1:**
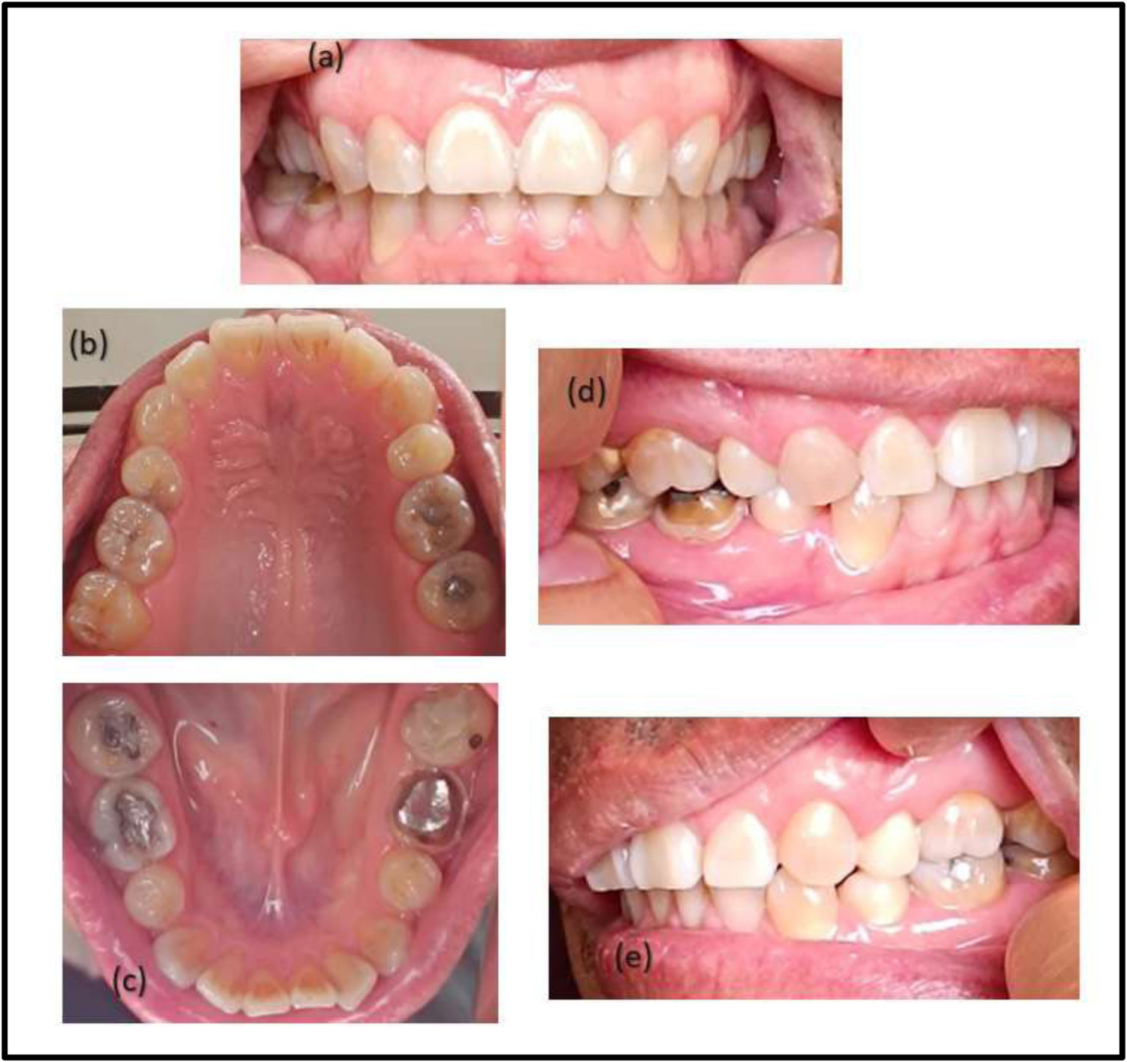
Example of intraoral images taken by the patient's family member: **(a)** frontal view; **(b)** upper occlusal view; **(c)** lower occlusal view; **(d)** right lateral view and **(e)** left lateral view.

The study population was comprised of adults who attended dental care at the dentistry service of the Polyclinic Chincha-EsSalud in Lima, Peru, from December 1, 2022, to March 30, 2023, being the inclusion criteria that these people should be 18 years old or more, with at least 20 permanent teeth in the mouth and that they had accepted the informed consent, excluding people with difficulties for opening the mouth or articular problems, with orthodontic treatment and some type of removable dental prosthesis. The number of teeth needed was calculated considering a 95% confidence level, 5% accuracy, 85% sensitivity, 90% specificity (15), and 90% prevalence of caries (19), requiring a minimum sample size of 1,383 teeth. An adjustment for possible losses of 20% was made, totaling 1,740 teeth. Considering that each patient should have at least 20 permanent teeth, 87 patients were selected, and the unit of analysis was the permanent dental piece of the adult subject of the study. The sample was selected through consecutive cases of those attending the dental service until the required size was reached (Figure 1).

Prior to the study, training and calibration of the examiners were conducted regarding the clinical and photographic diagnostic criteria. The training was carried out on 5 patients, and the assessment of inter-examiner agreement was performed on 12 patients, yielding kappa values of 0.80 for the clinical evaluation and 0.88 for the photographic evaluation.

### Clinical-visual diagnosis method

Each participant underwent a clinical diagnosis of dental caries by two trained and calibrated dentists, one of whom was a specialist dentist with more than 20 years of experience (gold standard); they spent an average of 10 minutes per patient. The evaluation was done on the patient lying in the dental chair, with artificial light, and using a plane mirror and exploratory probe. A tooth was classified as healthy or carious, considering the WHO diagnostic criteria (20), because it was a brief index to apply, effective for clinical and research purposes, and valid in comparison with other diagnostic indexes.

Codes 0 and 1 were used, as follows:

• Code 0: Healthy tooth and restored tooth without caries.
• Code 1: Any visible carious lesion, including active non-cavitated initial caries and arrested caries, as well as restored teeth with caries.

All visible surfaces of the crown were evaluated, considering the clinical crown rather than the root surface. Permanent teeth with single or multiple fixed rehabilitation (inlays, crowns, bridges, veneers, etc.) were excluded from this evaluation.

The clinical evaluation was performed in compliance with the biosafety protocols established by the Ministry of Health (21).

For calculating sensitivity and specificity, the record made by the gold standard dentist was considered.

### Diagnosis of dental caries through the smartphone-based remote photographic method

Previously, a smartphone was selected that was presented as one of the most used and easily accessible (Xiaomi Redmi 9A) with the Android operating system; 32 GB of capacity and 2 GB of RAM; and a 13 MP (megapixel) rear camera with f2.2 focus/aperture, HDR (high dynamic range), built-in AI (artificial intelligence), and single-tone flash. The images captured with this camera for this study were in photo mode. To ensure standardization, the same mobile device was used for all photographic shots.

During the clinical care visit, the patient's closest relative was given a video with the protocol for taking photographs of the oral cavity using the assigned smartphone model for 10 minutes with one focus and in automatic configuration. Afterward, a 20-minute training process was carried out to demonstrate their skills in taking photographs of the process. Fifteen intraoral images were taken per patient which included three of anterior view, three of superior occlusal view, three of inferior occlusal view, three of right lateral view, and three of left lateral view, as it shows in Figure 1. One of the researchers evaluated the centering of images, balanced resolution, and visualization of all the teeth (complete images), focused and in high-quality mode, discarding those images that did not meet the aforementioned criteria, selecting one photographic shot from each sector until obtaining the five shots corresponding to one patient. If the family member did not achieve a good quality photographic set, the training process was repeated once more. If a good photographic set was not achieved in this second opportunity, the patient was excluded. The selected images were stored in the internal memory of the smartphone and were downloaded through a secure internet connection to a computer.

The photographs were taken with the patient sitting on a dental stool and in a room illuminated with artificial light. The flash function was activated on the smartphone device, and a 2,5 × 3,3-inch handheld mirror was used for the patient. The patients were asked to retract their cheeks with their fingers (index and middle fingers); no cheek retractor or other auxiliary instrument was used for the photographic acquisition.

The photographic evaluation of the dental caries diagnosis was carried out by two independent evaluators (dentists), who were blinded to the visual evaluation previously performed. Both evaluators were trained and calibrated following the same criteria as the clinical visual assessment. One of them is a specialist dentist with more than 20 years of experience. Each evaluator recorded the information in a form specially designed for the study, which was done two weeks after the clinical visit.

For the photographic evaluation, the same diagnostic criteria as those used in the clinical assessment were considered. Additionally, due to the provision of a two-dimensional image, proximal surfaces were not taken into account.

Cases that were inconclusive in the photographic diagnosis were classified as healthy teeth (code 0).

The web-based image and data visualization application Google Photos was available for the photographic evaluation. Each evaluator reviewed the oral images and selected one image for each type of view which were in focus; in addition, the largest number of tooth surfaces were observed and with good resolution (22). Intraoral photographs were observed with normal magnification, macro settings, and a real color range after completing the review. Those teeth that could not be visualized in the photograph were not considered for the analysis.

### Statistical analysis

The descriptive analysis of dental caries for the proposed study method was performed using frequency distribution tables. In addition, Cohen's Kappa index was determined for estimating interobserver reliability in clinical and photographic evaluation. The results were interpreted according to the Landis and Koch rating scale, considering 0.01–0.20 as null or minimal, 0.21–0.40 as low, 0.41–0.60 as moderate, 0.61–0.80 substantial-good, and 0.81–1 almost perfect agreement (23). The tests were performed at a 5% significance level using the SPSS 26.0 statistical program.

The clinical visual diagnosis was considered the gold standard method. Sensitivity, specificity, positive and negative predictive value, and 95% confidence intervals were obtained using only the data collected by the most experienced evaluator at the clinical and photographic levels, respectively. The statistical program Epidat 3.1 was used. These measures were used to determine the diagnostic validity regarding dental caries since a nominal scale (present-absent) was used in this work.

## Results

Fifteen patients out of the 102 eligible ones were excluded because they did not agree to sign the informed consent or did not attend with a close relative to perform the process, so the final sample consisted of 87 individuals, 52 (59.8%) women and 35 (40.2%) men. The mean age was 49.06 years (SD = 15.36), with a minimum age of 19 years and a maximum age of 75 years.

**Figure 2:**
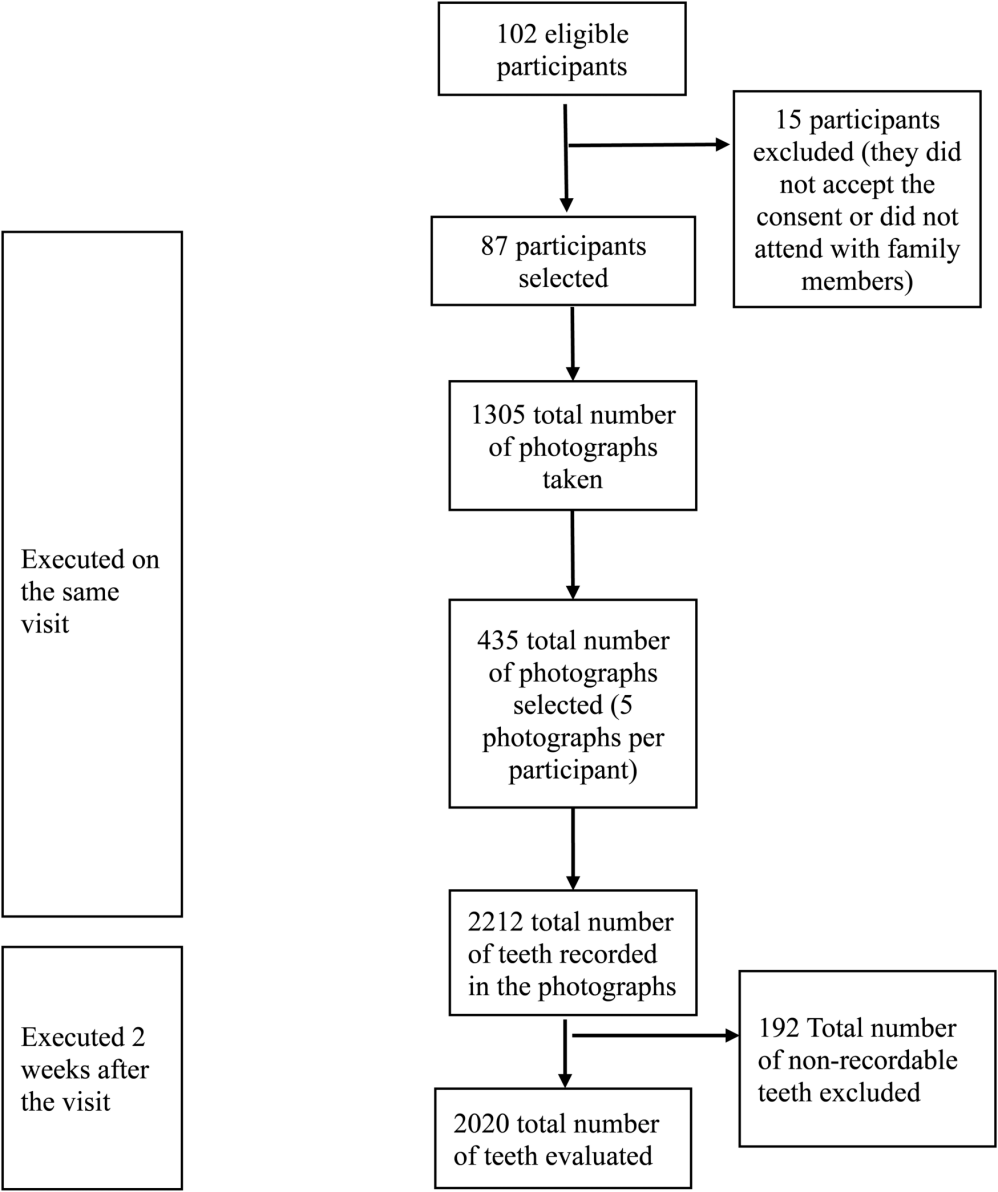
Flow chart of the smartphone-based photographic method for the detection of dental caries.

A total of 2,212 teeth were registered, of which 192 (8.7%) were excluded because they were not recordable for diagnosis, obtaining a total sample size of 2,020 teeth (Figure 2). There were 928 (45.9%) teeth with dental caries and 1,092 (54.1%) healthy teeth (Table 1).

**Table 1:** Frequency distribution of the diagnosis of dental caries in the clinical and photographic evaluation methods.

Evaluation methods	Clinical evaluation			
		Caries	Healthy	Total
Photographic evaluation	Caries	837 (41.4%)	53 (2.7%)	890 (44.1%)
	Healthy	91 (4.5%)	1,039 (51.4%)	1,130 (55.9%)
	Total	928 (45.9%)	1,092 (54.1%)	2,020 (100%)

### Diagnostic performance

In general, a high percentage of sensitivity was observed in the photographic evaluation (90.19%) and high specificity (95.15%). The positive and negative predictive values were 94.04 and 91.95, respectively (Table 2).

**Table 2:** Sensitivity, specificity, and predictive values of the photographic evaluation of dental caries.

Measurements	Value	IC 95%
Sensitivity	90.19	88.23–92.16
Specificity	95.15	93.83–96.47
Predictive value +	94.04	92.43–95.66
Predictive value -	91.95	90.32–93.58

The interexaminer agreement was examined both between the two clinical examiners and between the two photographic examiners concerning the caries diagnosis. An almost perfect agreement was observed, with kappa values of 0.935 (95% CI: 0.892–0.979) and 0.974 (95% CI: 0.930–1.00), respectively (Table 3).

**Table 3:** Interexaminer agreement in clinical and photographic evaluations.

Interexaminer agreement	*n*	Kappa	CI 95%	*P* Value
Clinical evaluation	2020	0.935	0.892–0.979	<0.001
Photographic evaluation	2020	0.974	0.930–1.00	<0.001

## Discussion

This study aimed to take advantage of the access that the general population has to the use of mobile devices and to give it a different approach since, at the dental level, few tools allow a diagnostic use in dental caries, which is important to consider because of the ease and practicality of the method (13, 24). The results of this research suggest that the photographic method using a smartphone offers a valid and reliable means for detecting dental caries, presenting high sensitivity and specificity concerning the clinical visual examination performed. It is important to consider that it was performed in an adult population with access to health services.

Regarding the high sensitivity found, we consider that the ability to discriminate incipient carious lesions from healthy teeth is somewhat confusing for some clinicians (11), obtaining in some cases moderate to low values (11, 25–28); but, currently, there are mobile devices with greater resolution and sharpness, which allows differentiating a healthy tooth from one with an initial lesion despite the presence of confusing factors such as saliva, blood or dental tartar (24, 29). Another aspect to highlight regarding the sensitivity found was the consideration in the analysis of teeth with restorations in poor condition with recurrent and incipient caries, which constituted an advantage due to the fewer false-negative errors. In addition, it is important to recognize the participation of expert dentists as examiners in this study (11, 30–32). This contrasts with other studies in which other professional groups or personnel in training participate, lacking the necessary experience for diagnosing dental caries (14, 30, 33). It is relevant to highlight that the participant's relative followed the photographic work protocol while taking the photographs, like previous experiences involving non-experts in the process, without specific knowledge of oral photography (26, 32, 33).

This study found a sensitivity level within the WHO reference standard of 0.85–0.90 (20), obtaining quite promising results with this technique for diagnosing carious lesions, which agrees with other studies where high sensitivity values were also found (30, 32, 34).

Concerning specificity, high values were also obtained, which could be explained by the ability of trained and calibrated dentists to differentiate with greater discernment between healthy teeth and teeth with mild caries (28, 30, 32). In the present study, we found concordant data (presenting fewer false positive discrimination errors) with similar values found by other authors (14, 15, 26, 34), although not as high as those reported in deciduous dentition by Al Shaya et al. (27).

High PPV (positive predictive value) and NPV (negative predictive value) values were found, which justifies the accuracy of the sensitivity and specificity scores obtained, similar to data obtained by other researchers, such as Al Shaya et al., with values above 90% in both cases (11, 27, 32).

In previous research, indicators of low to moderate agreement between evaluators for the detection of dental caries in permanent teeth were observed (11, 13, 14, 25, 33), probably due to the difficulties associated with the use of two-dimensional images that limit the identification of interproximal lesions, more complex caries indexes and the lower sharpness of the images. However, by using the more simplified WHO caries index (19) with improvements in the resolution of the photographic images and the greater diagnostic capacity of trained professionals, it can be affirmed that high levels of concordance were achieved between clinical and photographic evaluators. This method seems practical and capable of differentiating the different oral conditions (healthy-carious). It is relevant to point out that the photographic method exhibited a higher level of concordance than the clinical method, suggesting that the photographic evaluation is reliable and safe, consistent with other observed studies (15, 29, 32, 34).

Regarding the limitations of the present study, it was identified that the photographic analysis of the crown of the tooth, rather than the dental surfaces, does not allow for an adequate diagnosis of interproximal carious lesions, root lesions, or non-cariogenic cervical lesions (26). Furthermore, this method does not permit the recording of the severity of carious lesions (9). These limitations arise from the two-dimensional nature of the images, which does not allow for capturing these characteristics. Additionally, it is important to note that photographic recording requires a smartphone capable of taking photographs, storing them, and sending them for subsequent analysis, as well as internet connectivity, which may be limited in certain areas (34). Even you can find a hand mirror in a supermarket, it is also crucial to consider access to handheld mirrors for intraoral photography, which have variable dimensions and must fit intraorally to facilitate the respective photographic capture.

This study revealed several advantages, highlighting the performance of clinical dentists, who are experts in clinical and visual examination (13, 25, 34). This approach allows a more accurate diagnosis of carious lesions in comparison with less trained health professionals, who may have a higher margin of error (14, 15). In addition, we sought to replicate the conditions that patients face at home, where the lack of specific equipment for oral photographic capture and a profound lack of knowledge about photographic records is common. In this context, the patients’ relatives took the photographs, previously instructed through explanatory videos, using a conventional smartphone easily accessible to the population (33).

This approach is presented as a practical diagnostic tool, especially beneficial in teledentistry similar to what was found by Aly et al., where it can mitigate communication challenges and limited access to health services (28). Furthermore, by using an inexpensive and easily accessible device, this method facilitates the efficient recording, storage and sending of relevant information between users and health professionals.

## Conclusions

The use of the photographic method with smartphones for the diagnosis of dental caries has been confirmed as a valid and reliable technique in the detection of caries in adult permanent dentition. This approach appears as a practical diagnosis tool, especially beneficial in teledentistry, where it can mitigate communication challenges and limited access to health services. In addition, by using an inexpensive and easily accessible device, this method facilitates the efficient recording, storage, and sending of relevant information between users and healthcare professionals.

## Data Availability

The datasets presented in this study can be found in online repositories. The names of the repository/repositories and accession number(s) can be found below: Zenodo repository, https://zenodo.org/doi/10.5281/zenodo.10580117.
